# Modified Ride-On Cars and Young Children with Disabilities: Effects of Combining Mobility and Social Training

**DOI:** 10.3389/fped.2017.00299

**Published:** 2018-01-15

**Authors:** Hsiang-Han Huang, Yi-Mei Chen, Hsuan-Wen Huang, Ming-Ke Shih, Yu-Hsin Hsieh, Chia-Ling Chen

**Affiliations:** ^1^Department of Occupational Therapy, Graduate Institute of Behavioral Sciences, Chang Gung University, Taoyuan, Taiwan; ^2^Department of Physical Medicine and Rehabilitation, Chang Gung Memorial Hospital, Linkou, Taiwan; ^3^Department of Physical Medicine and Rehabilitation, Chang Gung Memorial Hospital at Taoyuan, Taoyuan, Taiwan; ^4^Department of Rehabilitation, Saint Mary’s Hospital Luodong, Yilan, Taiwan; ^5^Department of Physical Medicine and Rehabilitation, Chang Gung Memorial Hospital, Linkou, Taiwan; ^6^College of Medicine, Graduate Institute of Early Intervention, Chang Gung University, Taoyuan, Taiwan

**Keywords:** modified ride-on cars, mobility, social function, parenting stress, young children with disabilities

## Abstract

**Background:**

Research has shown that the use of power mobility devices is safe and beneficial for motor and cognitive development in children with motor disabilities; nevertheless, strong evidence of the benefits for social skill development is limited. This study aimed to examine the effects of combining ride-on car training with an adult-directed, social interaction program in a hospital-based environment on mobility and social functions in young children with motor disabilities.

**Methods:**

This study used a prospective, nonequivalent pretest–posttest control group design. Twenty-nine young children with motor disabilities, aged between 1 and 3 years, were recruited from local hospitals in Taiwan. The treatment group (*n* = 15) underwent 2-h ride-on car training sessions twice per week for a total of 9 weeks in the hospital environment. The control group (*n* = 14) underwent a 9-week home education program (mean: 200 min/week) focusing on mobility and social skills training. The Chinese version of the Pediatric Evaluation of Disability Inventory, Parenting Stress Index, and Goal Attainment Scaling were administered to all participants before and after the intervention, and at the end of the 9-week follow-up phase.

**Results:**

Mobility and social functions significantly improved in both groups after the 9-week intervention, but this improvement was not maintained at the follow-up phase. The treatment group showed significantly better improvement in social function, parenting stress levels, and goal achievement than the control group at posttest.

**Conclusion:**

This two-group design study showed the benefits of combining a ride-on car use with a family-centered, structured, social interaction program for positive impacts on mobility, social function, and parenting stress levels. The combination of a modified ride-on car and a social training program has the potential to enhance socialization in young children with motor disabilities.

**Clinical Trial Registration:**

www.ClinicalTrials.gov, identifier NCT02527499.

## Introduction

Independent mobility has been proven to improve motor, social, emotional, language, cognitive, and perceptual development in young children with typical development (1–3). According to the ecological theory, independent mobility guides a child’s perception to explore the surrounding environment and perceive relevant information through the perception–action cycle (2, 4). In the context without social interaction, independent mobility plays an important role in exploring the environment (e.g., being aware of heights, avoiding obstacles, and hiding) (2, 5). When becoming more social, children with independent mobility have more opportunities to interact with their parents or peers and participate in quality play (e.g., moving with peers, actively sharing toys, and initiating play with peers) (6, 7). Independent mobility increases opportunities for social participation, which facilitates their social development (8–10).

Young children with motor disabilities and limited independent mobility are at risk of secondary impairments, such as cognitive delay and atypical social function (11–13). Literature has shown that the use of power mobility devices (PMDs) is safe and beneficial for motor and cognitive development in children with motor disabilities younger than 3 years (14–16). Livingstone and Paleg (17) suggested that power mobility may address secondary effects, such as impaired socialization in very young children who cannot move and explore independently; nevertheless, evidence of the benefits for social skill development is limited and weak. Studies have found that the novel application of modified ride-on toy cars in home- or hospital-based environments might enhance independent mobility, motivation, and social function in young children with motor disabilities (18–21). However, Huang and Chen (18) found no significant differences between a ride-on car training and regular therapy group, although social functioning increased significantly in the ride-on car group.

Other studies have also reported that strategies allowing mobility and socialization intervention were important for improving motor and social functions in young children with disabilities (12, 22, 23). Evidence has suggested the combination of ride-on car training with a structured, adult-directed, social interaction program should be considered and may be beneficial for promoting socialization in various environments (17, 18, 23). In addition, caregivers’ involvement in the ride-on car training program may also help to decrease caregivers’ stress and provide an appropriate learning strategy for children (20, 21, 24). A similar concept applied in a family-centered service, which emphasizes coaching and cooperating with caregivers, increases treatment effects and helps participants to generalize learned skills to their natural environments (25). It is believed that the application of a family-centered approach in early intervention (EI) programs enhances functional performance of the child and caregivers’ empowerment (i.e., understanding their children better and having better control of the children) (26). Previous studies have integrated the caregiver’s role in ride-on car training and suggested the use of standardized measurements to assess outcomes of goals set by the family (i.e., Goal Attainment Scaling) (18–20, 27). Although some findings support the notion that ride-on car training may decrease parenting stress (18, 20, 21), these results must be interpreted with caution due to the lack of a control group with similar treatment dosage and mobility and social function goal setting.

To date, no study has examined the effects of using ride-on cars with the combination of structured, adult-directed, social interaction program on improving mobility, and social functions in young children with motor disabilities. The long-term effects of the ride-on car training on these functional outcomes also remain questionable. Therefore, the aim of this study was to examine the effects of combining ride-on car training with a social training program in a hospital-based environment on mobility, social function, parenting stress levels, and goal achievement in young children with disabilities and their families. This study also included a 9-week follow-up phase and a control group that involved home education program with similar treatment dosages and goal settings. We hypothesized that the ride-on car training group would show more benefits in mobility, social function, parenting stress, and goal achievement compared with the home education group, and that the combined use of ride-on car training and a social skill training program is an alternative treatment for enhancing psychosocial function in young children with motor disabilities.

## Methods

This study used a prospective, nonequivalent pre–posttest control group design (28). The study duration for each participant was 18 weeks. Assessments took place on three occasions in the assessment room at a university: before and after the 9-week intervention, and at the end of the 9-week follow-up phase. One licensed occupational therapist (OT) blinded to the study hypotheses was responsible for treatment. Assessments were performed by another licensed OT who was not involved in the treatment or data collection and was blinded to which group the children belonged. The participants were recruited from Taoyuan and Linkou Chang Gung Memorial Hospitals and the community (e.g., health-care practitioners or self-referrals) in Taoyuan and Taipei, Taiwan.

### Participants

Participants in the study were 29 young children with disabilities aged between 1 and 3 years, including a group who received ride-on car training combined with an adult-directed, social interaction program (*n* = 15) and a group who received a home education program (*n* = 14). The inclusion criteria included the following: (1) motor delays (SD > 1.5), assessed according to the Chinese Child Development Inventory (29, 30) in the Early Intervention Child Development Joint Evaluation Center (providing relevant developmental assessments to determine the child’s developmental status and if the child requires EI services), resulting in motor impairments that prevented functional independent mobility, such as rolling, crawling, and walking; (2) age between 12 and 36 months old; (3) the ability to tolerate sitting with support for 30 min; (4) the ability to reach objects with either one or two hands; and (5) approval of the caregivers to participate at Taoyuan Chang Gung Memorial Hospital. The exclusion criteria included the following: (1) young children with severe sensory impairments, such as blindness or deafness and (2) caregivers were not able to make a time commitment for the training phase. This study was reviewed and approved by the Chang Gung Medical Foundation Institutional Review Board. This trial was registered in the www.ClinicalTrials.gov: NCT02527499. The parents provided written informed consent before their children participated in the study.

### Procedure

**Figure 1:**
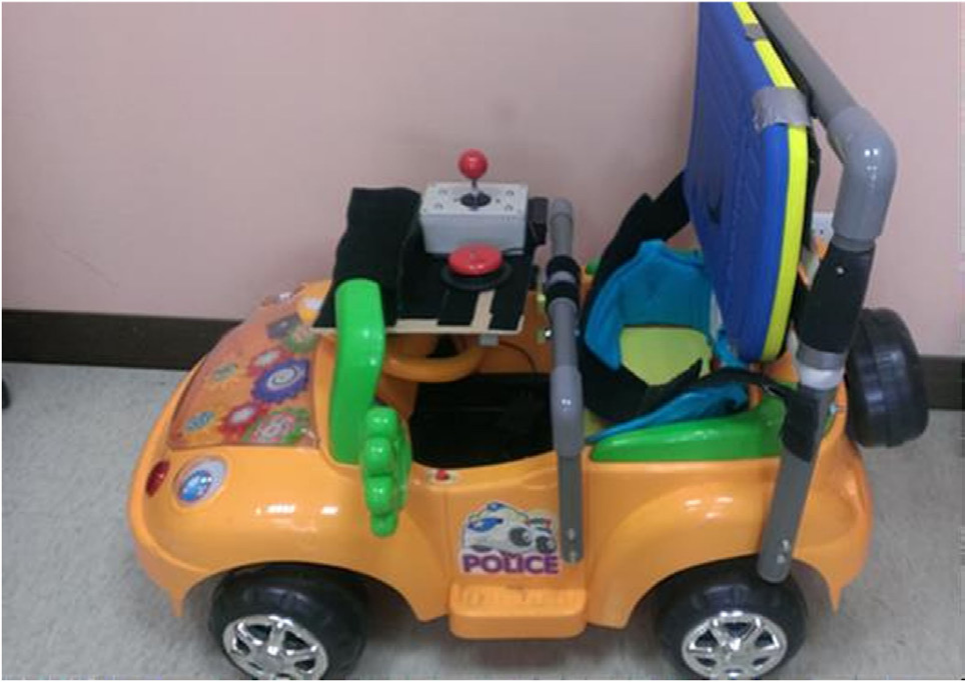
The modified ride-on toy car.

The children were divided into two groups: the treatment group (a hospital-based, ride-on car use combined with social training program) and the control group (home education program without the use of a ride-on car). This was not a randomized controlled trial due to a practical reason: the research team wanted to provide treatments for the participants within a reachable geographical area and in compliance with the treatment schedule. Once the participant was enrolled in the study and assigned to a treatment group, the research team modified a toy car based on each participant’s capabilities during the pre-intervention phase, e.g., seat and steering wheel modifications. The control system included switch and joystick activations (Figure 1) as used in the previous studies (18, 19). The caregivers or therapist could use a wireless joystick as a shared controller to provide assistance in making turns and participate in the interactive training program without stopping or interrupting the child’s driving (31). Participants in both groups continued their regular therapy throughout the whole study, including physical, occupational, and speech therapies.

#### Intervention

##### Treatment Group (Ride-On Car Training Program)

The ride-on car training sessions were conducted at Taoyuan Chang Gung Memorial Hospital using public spaces (e.g., hallways, garden, and food court). The training program was developed based on the previous studies (18, 23). The research team and caregivers discussed the training goal with the OT who conducted the program for 2 h/session, 2 sessions/week for a total of 9 weeks. The 2-h intervention session included the following: (1) natural play as a warm up activity (15 min); (2) mobility and social training without cars (30–45 min); and (3) mobility and social training with cars (60–75 min). All the details of treatment sessions were based on the expected independent mobility and social function goals set by caregivers in the Goal Attainment Scaling (GAS) after the pretest. The 30–45 min individualized treatment program involved motor skills training related to mobility and social impairments, such as postural stability, postural control, and social engagement. Simultaneously, the caregivers learned how to identify their child’s difficulties and how to apply remedies at home. The subsequent 60–75 min driving session involved the participants learning cause–effect concepts by driving the toy car (i.e., pressing the switch for moving and releasing it for stopping). The therapist and caregivers then used verbal prompts to encourage children to drive and explore the surrounding environment.

An adult-directed social training principle (23) was integrated into the car play session. The social-interactive activities were graded based on the participants’ current social function. First, one-on-one instruction was used to teach participants how to perform desired social behaviors (e.g., greeting, gestures, or vocalizations) using demonstrations and physical guidance. Second, the therapist and caregivers guided the participant to perform desired social behaviors while meeting people in the hallway or in the stores. Encouragements were expressed explicitly and loudly to compliment the participant’s achievement as positive feedback. Finally, participants were directed to accomplish social interaction tasks, such as sharing toys with other adults or children he/she met during driving or collecting stickers from employees in the stores. In addition, to enhance parent–child relationships, several games were developed to provide opportunities for practicing social interaction with family members (e.g., hide and seek, peekaboo, “I see you,” and role play games).

##### Control Group (Home Education Program)

The control group received a Chinese educational booklet with general guidelines for increasing their child’s motor and social functions at home. The booklet included guidelines in three main domains: socialization, mobility, and motivation, which were the goals of this home program. Each guideline was graded with different activities and the general principle was similar to the treatment group, which focused on performing desired social behaviors, providing encouragement, and accomplishing social interaction tasks. The therapist would set up goals with the parents through the use of the GAS and provide advice to the caregivers on which activities were appropriate to start with after the pretest. The therapist also suggested a treatment intensity of 35 min/day, 7 days/week during the 9-week intervention. The therapist called the caregivers every week to answer relevant questions, provide suggestions and confirm compliance. The caregivers kept an activity log, including home training duration and their child’s emotional reactions. The participants continued their regular therapy throughout the whole study, including physical, occupational, and speech therapies.

#### Follow-up

This period involved 9 weeks following the above treatment programs, during which time no treatment programs were delivered to the participants except for their own regular therapy.

#### Assessment

The Chinese version of the Pediatric Evaluation of Disability Inventory (PEDI-C) (32), which quantifies self-care, mobility, and social function in children aged 6 months to 7 years, was used to examine the mobility and social functions in both groups (33). The PEDI-C is a parent report and is a reliable and valid assessment tool, which is especially useful for tracking changes in functional skills (32, 33). Each domain can be used separately (34, 35). For the purposes of this study, we have only shown the scaled scores for the functional skills section of mobility and social domains in Section “Results.” The scaled scores vary within the range of 0–100; the higher the score is, the higher the difficulty level of the task the child could perform. The scaled scores enable the comparison of children’s abilities on the same scale regardless of age (36). The change in PEDI-C scores using 11 as the cutoff point determines the clinically meaningful and functional changes (37).

Parenting stress level and goal achievement were assessed using the Parenting Stress Index Short Form (PSI/SF) and Goal Attainment Scaling (GAS). The PSI is a tool with excellent validity and reliability (0.55–0.80) (38) designed to measure the overall level of parenting stress experienced by parents/caregivers of children aged between 1 month and 12 years. In addition, GAS is a family-centered, criteria-referenced, responsive tool (39). It has good validity and excellent inter-rater agreements with ICCs of 0.90 or above (40, 41). The therapist set functional goals with the client and divided the goal into five grades: −2 (current level of performance); −1 (less than desired outcome); 0 (desired outcome) to +1 (greater than expected outcome); and +2 (much greater than expected outcome). The raw score can be converted into *T*-scores to see if the participant achieved their expectations. A *T*-score of 50 indicated that the participant accomplished the desired outcome.

In addition, we used an activity log to record the child’s performance, treatment duration, his/her emotional reactions, the family’s perceptions on the training program, and its’ effects on play and family interactions every week during the intervention phase for both groups. The activity log was mostly qualitative data that involved parents’ descriptions except for the treatment duration every day.

### Statistical Analysis

Normality of the data was examined using Kolmogorov–Smirnov and Shapiro–Wilk tests (42). Due to the normal distribution of the data, the independent *t*-test and chi-square test were used to compare demographic data in the two groups. The effects of the two programs were evaluated using repeated measures analysis of variance [group (2) × time (3)] with the outcome measures as the dependent variables, except for the GAS scores. The overall interaction (group × test session) allowed testing of whether the time course differed between groups. In the case of a significant interaction, time and treatment effects were further analyzed using the pairwise *post hoc* Bonferroni test separately at each time point with *p* value of 0.017. Due to the two GAS sessions in two groups (pretest and posttest), we used paired and independent *t*-tests to compare the mean differences of goal achievement within and between groups. An α level of 0.05 represented a significant difference. All analyses were performed using Statistical Package for Social Science version 22.0 software (SPSS Inc., Chicago, IL, USA).

## Results

### Participants

The demographic data of the 29 young children are shown in Table 1, including age, sex, diagnosis, height, weight, and the amount of regular therapy and additional therapy received each week. On average, the treatment group [15 participants, mean (SD) age: 18.53 (7.69) months] received 104 min/week of regular therapy and 240 min/week of ride-on car training, whereas the control group [14 participants, mean (SD) age: 18.14 (7.33) months] received 121.25 min/week of regular therapy and 200.33 min/week of home therapy. No significant difference was found between the treatment and control groups regarding these variables, including age, sex, and the amount of regular therapy and additional therapy received each week (Table 1).

**Table 1:** Demographic data.

	Treatment group (*n* = 15)	Control group (*n* = 14)	*t*	*p*
Mean age-month (SD)	18.53 (7.69)	18.14 (7.33)	0.14	0.89
Diagnosis, *n* (%)				0.32
Developmental delay	11 (74%)	9 (65%)		
Cerebral palsy	2 (13%)	2 (14%)		
Others	2 (13%)	3 (21%)		
Gender, *n*				0.56
Male	7 (47%)	5 (36%)		
Female	8 (53%)	9 (64%)		
Regular treatment time in minutes per week (SD)	104 (66.95)	121.25 (78.32)	−0.64	0.53
Ride-on car or home training time in minutes per week (SD)	240 (0)	200.33 (123.73)	0.75	0.07

### Mobility, Social Function, and Parenting Stress Levels

**Figure 2:**
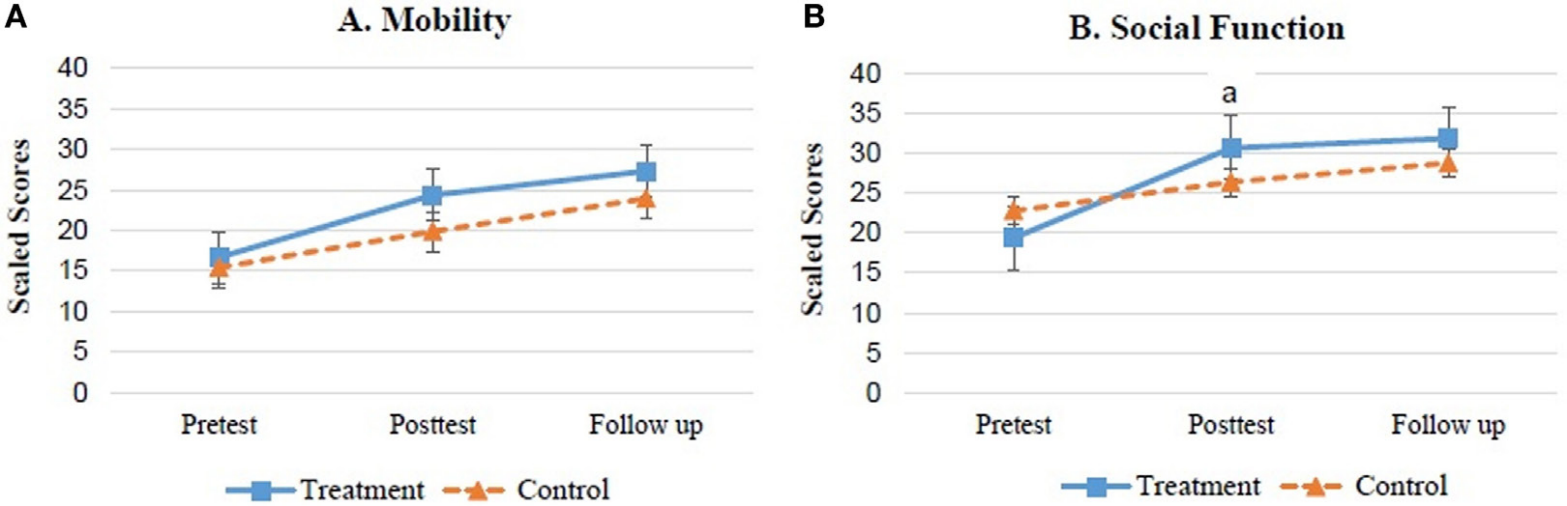
Mean ± SEM scaled scores of PEDI for both groups. **(A)** Mobility. **(B)** Social function. ^a^Difference is significant (*p* < 0.05) between two groups.

**Figure 3:**
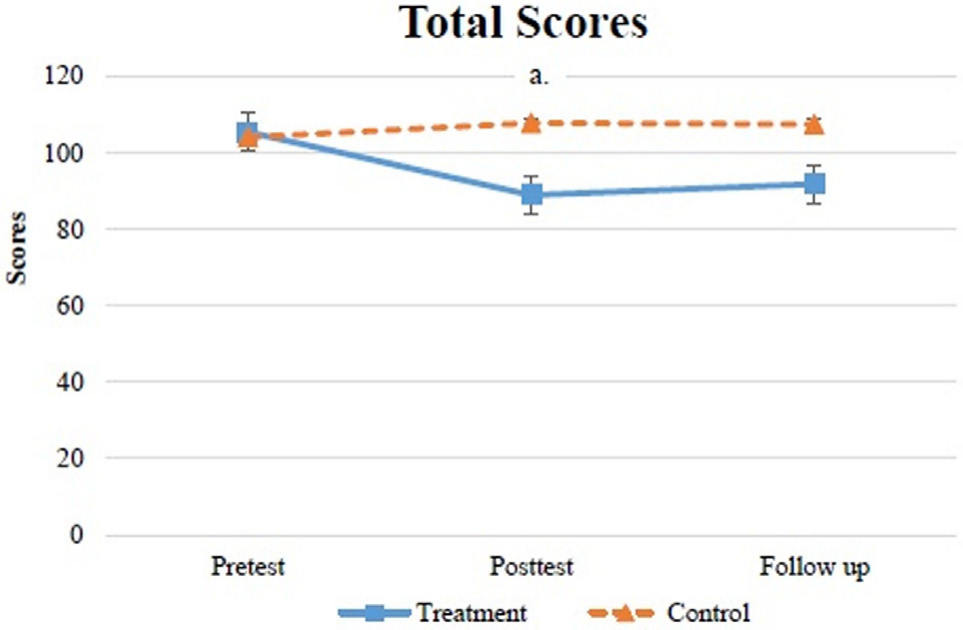
Mean ± SEM total scores of PSI for both groups. ^a^Difference is significant (*p* < 0.05) between two groups.

Table 2 and Figures 2 and 3 present the results of mobility, social function, and parenting stress levels assessed using the PEDI-C and PSI during pretest, posttest, and follow-up test. No significant differences were found between the treatment and control groups regarding mobility (*p* = 0.76), social function (*p* = 0.51), and caregiver stress levels (*p* = 0.86) during pretest. Table 2 shows the means for the treatment and control groups at each time point, i.e., the pretest to immediate posttest and posttest to follow-up differences for all measures. For the mobility function measured by PEDI, there was a 7.69 (5.86) and a 4.45 (7.52) increase for the treatment and control groups, respectively, at posttest (Figure 2A). A main effect of testing session was seen for both treatments and the *post hoc* test only showed a significant difference for pretest and posttest scores (*p* < 0.01).

**Table 2:** Comparisons on mobility, social function, and parenting stress levels between the two groups.

	Pretest, mean (SD) (95% CI)	Posttest, mean (SD) (95% CI)	Follow-up, mean (SD) (95% CI)	Group effect, *p* value (partial η^2^)	Testing session effect, *p* value (partial η^2^)	Interaction effect, *p* value (partial η^2^)
PEDI_Mobility	*p* = 0.48 (0.019)				*p* < 0.01 (0.414)	*p* = 0.58 (0.020)
Treatment (*n* = 15)	16.64 (10.99) (10.55, 22.73)	24.33 (11.49) (17.97, 30.69)	27.31 (13.99) (19.57, 35.06)			
Control (*n* = 14)	15.40 (10.48) (9.35, 21.45)	19.85 (12.08) (12.87, 26.83)	23.92 (14.22) (15.71, 32.13)			
PEDI_Social Function	*p* = 0.74 (0.004)				*p* < 0.01 (0.422)	*p* = 0.03 (0.123)
Treatment (*n* = 15)	19.35 (13.89) (11.66, 27.05)	31.21 (11.45) (24.87, 37.55)	31.81 (14.16) (23.97, 39.65)			
Control (*n* = 14)	22.73 (13.24) (15.08, 30.38)	26.34 (14.41) (18.02, 34.66)	28.71 (14.08) (20.58, 36.85)			
PSI_Total Scores				*p* = 0.11 (0.091)	*p* = 0.32 (0.042)	*p* < 0.01 (0.211)
Treatment (*n* = 15)	101.40 (21.13) (89.70, 113.10)	88.93 (22.50) (76.47, 101.39)	91.87 (19.17) (81.25, 102.48)			
Control (*n* = 14)	102.71 (18.85) (91.83, 113.60)	107.79 (22.26) (94.93, 120.64)	107.43 (21.68) (94.91, 119.95)			

*PEDI, Pediatric Evaluation of Disability Inventory; PSI, Parenting Stress Index*.

There was a significant time × group interaction for the PEDI social function (*p* = 0.03). The *post hoc* tests indicated that in both groups, children had a better social function score at the posttest and follow-up than in the pretest (*p* < 0.01). The social function scores improved significantly, with 11.85 and 3.61 points at posttest for the treatment and control groups, respectively (Figure 2B). The pairwise *post hoc* test showed significantly more treatment effects on social function for the ride-on car training program compared with the home education program (*p* = 0.01).

For parenting stress levels, a significant time × group interaction for PSI total scores was observed (*p* < 0.01). The *post hoc* tests indicated that in the ride-on car training group, the caregivers had significantly lower stress levels at the posttest than at the pretest (*p* = 0.01) (Table 2; Figure 3). In the control group, no significant difference was observed between the three testing sessions. In addition, pairwise comparisons indicated that the ride-on car training produced better effects on decreasing parenting stress than the home education program (*p* < 0.01).

### Goal Achievements

Both groups had significant improvements in goal achievements after receiving the 9-week intervention (treatment: *p* < 0.01, control: *p* < 0.01) (Table 3). However, only the treatment group exceeded the expected level of goal performance at posttest (*T*-score of 50). Furthermore, the ride-on car training group made greater progress than the home education group, as indicated by significant group differences in their *T*-score at posttest (*p* < 0.01).

**Table 3:** Comparisons on Goal Attainment Scaling (GAS) between the two groups.

	Pretest, mean (SD) (95% CI)	Posttest, mean (SD) (95% CI)
Treatment (*n* = 15)	22.54 (7.83) (18.20, 26.88)	62.11 (10.39)^a^^,^^b^ (56.35, 67.86)
Control (*n* = 14)	21.80 (1.68) (20.79, 22.82)	43.99 (13.93)^b^ (35.57, 52.40)

*^a^Difference is significant (*p* < 0.05) between two groups*.

*^b^Difference is significant (*p* < 0.05) within the group*.

### Self-Developed Activity Log

The family’s compliance of ride-on car and home education program remained high throughout the intervention phase. The treatment group had the intensity of 240 min/week for a total of 9 weeks during intervention. From the activity log, the duration of home education group varied from 0 to 50 min/day (mean 200.33 ± 123.73 min/week) due to a child’s or a family’s condition, e.g., sickness or vacation. Twelve participants of the treatment group (80%) and seven participants of the control group (50%) provided detailed descriptions regarding their children’s performances and emotions during the intervention. From their feedback, the caregivers of the treatment group reported that their children had more self-initiated movements, improved cognition, and were willing to interact with family members more than before. The caregivers of the control group reported that their children tended to stay in the same place for the first 5 weeks and they spent much time encouraging them to move and play. Children started to move more after 5 weeks; however, they still tended to sit or lay on the floor to play overall.

## Discussion

The use of modified ride-on toy cars as a type of PMD has become an innovative, alternative option to enhance early independent mobility and psychosocial function in young children with disabilities. These cars are light weight, low cost, customized, and attractive compared with power wheel chairs (24, 43). This is the first two-group design study that included a 9-week follow-up phase and examined the effects of combining ride-on car training with a family-centered, adult-directed, social interaction program on mobility and social functions in young children with disabilities in the hospital environment. The findings showed significant improvements in mobility and social functions in both the ride-on car training and home education groups after a 9-week intervention. In particular, the ride-on car training group had significantly more positive changes with regard to social function than the home education group. In addition, with significantly decreased parenting stress levels and increased goal achievement scores, the advantages of integrating this family-centered service concept into treatment programs are clear. The combination of ride-on car use with a family-centered, structured, social interaction program in the hospital environment is feasible and beneficial for facilitating mobility and social functions in young children with disabilities.

A ride-on toy car is an assistive device for young children with disabilities to travel around (24, 43). Combined with a social skill training program, it provides increased opportunities for socialization and varied contexts that may help to promote acquisition of social skills (23, 44). The current results on mobility and social functions are consistent with previous studies, which emphasize the benefits of PMDs to assist young children with disabilities to move freely, be more active in their environment, and learn the consequences of their actions (14, 18, 19, 21, 45). The treatment effects are also supported by feedback obtained from the caregivers in the treatment group. They reported on our self-developed activity log that children became more active and their self-initiated movements increased after gaining independent mobility with the ride-on cars. By contrast, according to the responses provided by caregivers in the control group, half of the children did not show any intention to move during the first 5 weeks and the caregivers spent the most time encouraging and teaching the child how to move.

Although the caregivers’ feedback from the two groups was different, we found similar effects on mobility function in the treatment and control groups. The dose–response relationship between ride-on car use and mobility function, and the inability to transfer the mobility skills from one context to another may be the two reasons relating to the current finding (23, 46). Ragonesi et al. (23) suspected that combining a structured, adult-directed, social training program might affect the progress of mobility function due to the focus on increasing socialization over mobility. Our finding of significantly more increased social function in the ride-on car training group provides evidence for this assumption. In addition, the lack of toy cars at home may partially explain that although the participants in the ride-on car training group were independent drivers in the hospital environment during intervention, they did not automatically transfer this level of mobility to daily life during follow-up. By contrast, participants in the control group may have continued their mobility skills practice during the follow-up phase due to the learned strategies from the educational booklets. The decreased change scores on mobility in the treatment group during the 9-week follow-up phase supports this speculation, while similar change scores were observed in the control group during the intervention and follow-up phases. With these outcomes and possible explanations, we propose that future research may identify the various “dose–response” relationships between the amount of mobility and social skills training and changes in the related functions during early PMD use combined with a social interaction training program.

In addition, Smith (47) found that combining family-centered service with social–emotional intervention could enhance the parent–child interaction and thus decrease caregivers’ stress levels. Our study results support this finding. In the ride-on car training combined with social interaction program, the therapist and the caregivers both developed some social games involving the ride-on car to provide the participants with opportunities to interact with family members (e.g., hide and seek, peekaboo, “I see you,” and role play games), which might have positive impacts on the quality of parent–child interaction and improve caregiver perceptions of their child’s capabilities. The significant improvements in social function and parenting stress observed in the ride-on car training group during the intervention, not the follow-up phase, provides evidence to support the effects of combining a family-centered services model with a ride-on car training program for improving social function of the children and lowering parenting stress of the caregivers. As a comparison, the control group also had the opportunities to discuss with the therapist once every week; however, the consultation time was less than the treatment group and the non-randomized recruitment method may relate to the heterogeneity of the participants regarding socioeconomic status and developmental levels, which may be the factors contributing to the observed outcome of parenting stress. The socioeconomic levels of the parents are identified as an important predictor of successfully completing such telephone-assisted intervention (48, 49). The lack of relevant information in the control group, i.e., actual consultation time, demographic data, and feedback from all the caregivers, may limit our in-depth discussion on this aspect. To increase the rigorous level of evidence, future studies should consider using randomized recruitment method and incorporating feasible tools, e.g., mobile technology over the Internet, to provide immediate feedback of treatment locations and cost, and quantified value of consultation time objectively (50, 51).

Of note is that only the ride-on car training group achieved expected functional goals and they had greater goal achievements than the home education group, verifying the benefits an integrated program for improving functional skills and family function in young children with disabilities, as suggested by previous studies (18, 19, 45). Studies report that limited mobility and social functions are the important factors relating to caregivers’ stress and the functional improvement in mobility may become a determining factor for lowering caregivers’ stress levels (11, 52). Combining the technique of family-centered service (i.e., coaching) into ride-on car training programs is beneficial for decreasing caregivers’ stress levels and achieving the expected family goals (18, 20, 53). The methods include teaching the caregivers to be sensitive to their child’s behaviors or performance and responding to them properly during intervention. Caregivers’ involvement in the ride-on car training program allows them to have discussion with the therapists, practice the techniques, express their opinions, and observe the behavioral changes in their children directly (18, 53). Although the control group had the opportunities to discuss with the therapist once every week and showed improvements, the consultation time was less than the treatment group, which may have impacts on the treatment effects. Future studies could combine assessment tools (e.g., the application of relevant questionnaires or semi-structured interviews) to examine parental perceptions of their child’s capabilities and daily function and its impacts on early PMD training.

### Limitations

There are some limitations in this study. First, our study suggests that the combination of ride-on car use and a structured, adult-directed, social program or home education program can be effective; however, we did not analyze the effect of frequency of mobility and social function training on the magnitude of treatment effects. Future studies should use an activity log to record durations of movement and social interaction to investigate the various dose–response relationships between ride-on car use and mobility and social functions. Second, the lack of control over the received therapy in the follow-up phase in the home education group may have contributed to the differences observed in this study. To examine the dose–response relationship discussed earlier, future studies should consider the relevant factors (dose, intensity, and environment) and design a matched, control group with no additional therapy provided in the follow-up phase. For example, a comparison with the same dose and intensity of ride-on car training and conventional therapy in the same location, e.g., physical therapy or occupational therapy focusing on mobility and social functions, may be applied to provide detailed information regarding the efficient treatments on mobility and socialization. Third, sample size restricted our ability to generalize the results to all young children with motor disabilities. In addition, non-randomized recruitment method may result in differences in developmental and socioeconomic levels between the two groups, which may further impact the observed outcomes (14, 17). A larger sample with different developmental and socioeconomic levels with a randomized controlled trial design is suggested to enable a complete and rigorous examination of this topic.

### Conclusion

Despite the increasing number of studies on using PMDs for enhancing mobility and overall development, there is still limited evidence on the impacts of independent mobility and psychological development in young children with disabilities (14, 27, 54). To facilitate participation in play in different environments, Livingstone and Paleg’s Delphi study (17) suggested that the development of inexpensive, light weight, child- and family-friendly PMDs is needed. Our findings show the significance of incorporating mobility device training with the social interaction programs to advance social function in young children with motor disabilities. Furthermore, the transition of service delivery from an expert-centered service to a family-centered service is an important concept to maximize the treatment effects. The overall findings of this study will provide clinicians a novel, low cost, and fun option to use in a hospital-based environment for improving young children’s mobility and social functions.

## Ethics Statement

This study was carried out in accordance with the recommendations of Chang Gung Medical Foundation Institutional Review Board with written informed consent from all subjects. All subjects gave written informed consent in accordance with the Declaration of Helsinki. The protocol was approved by the Chang Gung Medical Foundation Institutional Review Board.

## Author Contributions

H-HH is responsible for the design, data interpretation, drafting, and work revision. Y-MC is responsible for providing the interventions and drafting. H-WH is responsible for providing the assessments. M-KS and Y-HH are responsible for data management and analysis. C-LC is responsible for providing assistance in recruitment.

## Conflict of Interest Statement

The authors declare that the research was conducted in the absence of any commercial or financial relationships that could be construed as a potential conflict of interest.

## References

[1] Campos JJ Anderson DI Barbu-Roth MA Hubbard EM Hertenstein MJ Witherington D Travel broadens the mind. Infancy (2000) 1:149–219. 10.1207/S15327078IN0102_1 32680291

[2] Gibson EJ Exploratory behavior in the development of perceiving, acting, and the acquiring of knowledge. Annu Rev (1988) 39:1–41. 10.1146/annurev.ps.39.020188.000245

[3] Clearfield MW Osborne CN Mullen M. Learning by looking: infants’ social looking behavior across the transition from crawling to walking. J Exp Child Psychol (2008) 100(4):297–307. 10.1016/j.jecp.2008.03.005 18452944

[4] Gibson EJ Pick AD An Ecological Approach to Perceptual Learning and Development. New York: Oxford University Press (2000).

[5] Anderson DI Campos JJ Witherington DC Dahl A Rivera M He M The role of locomotion in psychological development. Front Psychol (2013) 4:440. 10.3389/fpsyg.2013.00440 23888146 PMC3719016

[6] Guerette P Furumasu J Tefft D. The positive effects of early powered mobility on children’s psychosocial and play skills. Assist Technol (2013) 25(1):39–48. 10.1080/10400435.2012.685824 23527430

[7] Smith PK Cowie H Blades M Early social behavior and social interactions. 5th ed Smith PK, editor. Understanding Children’s Development. UK: Wiley (2011). p. 95–9.

[8] Clearfield MW. Learning to walk changes infants’ social interactions. Infant Behav Dev (2011) 34(1):15–25. 10.1016/j.infbeh.2010.04.008 20478619

[9] Gustafson GE Effects of the ability to locomote on infants’ social and exploratory behaviors: an experimental study. Dev Psychol (1984) 20(3):397–405. 10.1037/0012-1649.20.3.397

[10] Gibson EJ Pick AD An ecological appraoch to perceptual development. In: Gibson EJ, editor. An Ecological Approach to Perceptual Learning and Development. New York: Oxford University Press (2000). p. 14–24.

[11] Chan HS Lau PH Fong KH Poon D Lam CC. Neuroimpairment, activity limitation, and participation restriction among children with cerebral palsy in Hong Kong. Hong Kong Med J (2005) 11(5):342–50. 16219953

[12] Jones MA McEwen IR Neas BR. Effects of power wheelchairs on the development and function of young children with severe motor impairments. Pediatr Phys Ther (2012) 24(2):131–40; discussion 40. 10.1097/PEP.0b013e31824c5fdc 22466379

[13] Fernandes T Independent mobility for children with disabilities. Int J Ther Rehabil (2006) 13:329–33. 10.12968/ijtr.2006.13.7.21410

[14] Livingstone R Field D. Systematic review of power mobility outcomes for infants, children and adolescents with mobility limitations. Clin Rehabil (2014) 28(10):954–64. 10.1177/0269215514531262 24764156

[15] Feldner HA Logan SW Galloway JC. Why the time is right for a radical paradigm shift in early powered mobility: the role of powered mobility technology devices, policy and stakeholders. Disabil Rehabil Assist Technol (2015):1–14. 10.3109/17483107.2015.1079651 26340446

[16] Wiart L Darrah J Changing philosophical perspectives on the management of children with physical disabilities – their effect on the use of powered mobility. Disabil Rehabil (2002) 24(9):492–8. 10.1080/09638280110105240 12097218

[17] Livingstone R Paleg G. Practice considerations for the introduction and use of power mobility for children. Dev Med Child Neurol (2014) 56(3):210–21. 10.1111/dmcn.12245 23998510

[18] Huang HH Chen CL. The use of modified ride-on cars to maximize mobility and improve socialization – a group design. Res Dev Disabil (2017) 61:172–80. 10.1016/j.ridd.2017.01.002 28087203

[19] Huang HH Chen YM Huang HW Ride-on car training for behavioral changes in mobility and socialization among young children with disabilities. Pediatr Phys Ther (2017) 29(3):207–13. 10.1097/PEP.0000000000000426 28654486

[20] Huang HH Ragonesi CB Stoner T Peffley T Galloway JC. Modified toy cars for mobility and socialization: case report of a child with cerebral palsy. Pediatr Phys Ther (2014) 26(1):76–84. 10.1097/PEP.0000000000000001 24263247

[21] Logan SW Huang HH Stahlin K Galloway JC. Modified ride-on car for mobility and socialization: single-case study of an infant with down syndrome. Pediatr Phys Ther (2014) 26(4):418–26. 10.1097/PEP.0000000000000070 25192001

[22] Ragonesi CB Chen X Agrawal S Galloway JC. Power mobility and socialization in preschool: a case study of a child with cerebral palsy. Pediatr Phys Ther (2010) 22(3):322–9. 10.1097/PEP.0b013e3181eab240 20699785

[23] Ragonesi CB Chen X Agrawal S Galloway JC. Power mobility and socialization in preschool: follow-up case study of a child with cerebral palsy. Pediatr Phys Ther (2011) 23(4):399–406. 10.1097/PEP.0b013e318235266a 22090084 PMC3266169

[24] Huang HH Galloway JC. Modified ride-on toy cars for early power mobility: a technical report. Pediatr Phys Ther (2012) 24(2):149–54. 10.1097/PEP.0b013e31824d73f9 22466382 PMC3324847

[25] Oono IP Honey EJ McConachie H Parent-mediated early intervention for young children with autism spectrum disorders (ASD). Cochrane Database Syst Rev (2013) 4:1–98. 10.1002/14651858.CD009774.pub2 PMC11831248 23633377

[26] Rosenbaum P King S Law M King G Evans J Family-centred service: a conceptual framework and research review. Phys Occup Ther Pediatr (1998) 18:1–20. 10.1080/J006v18n01_01

[27] Kenyon LK Farris JP Gallagher C Hammond L Webster LM Aldrich NJ. Power mobility training for young children with multiple, severe impairments: a case series. Phys Occup Ther Pediatr (2017) 37(1):19–34. 10.3109/01942638.2015.1108380 26735082

[28] Portney LG Watkins MP 3rd ed In: Mehalik C, editor. Foundations of Clinical Research: Applications to Practice. Upper Saddle River, NJ: Pearson/Prentice Hall (2015).

[29] Hsu CC Su S Shao SJ Lin CC Soong WT Chinese child developmental inventory: a tentative normative data. Acta Paediatrica Sinica (1978) 19:142–57.

[30] Wu HC Hsu CC Chiu V Yeh YJ Wen SH Diagnostic validity of the Chinese Child Development Inventory in screening children with developmental language delay. Tzu Chi Med J (2013) 25:228–32. 10.1016/j.tcmj.2013.07.004

[31] Mitchell IM Viswanathan P Adhikari B Rothfels E Mackworth AK, editors. Shared control policies for safe wheelchair navigation of elderly adults with cognitive and mobility impairments: designing a wizard of OZ study. Proc Am Control Conf. Portland, OR (2014).

[32] Chen KL Hsieh CL Sheu CF Hu FC Tseng MH. Reliability and validity of a Chinese version of the pediatric evaluation of disability inventory in children with cerebral palsy. J Rehabil Med (2009) 41(4):273–8. 10.2340/16501977-0319 19247548

[33] Feldman AB Haley SM Coryell J. Concurrent and construct validity of the pediatric evaluation of disability inventory. Phys Ther (1990) 70(10):602–10. 10.1093/ptj/70.10.602 2217539

[34] Knox V Usen Y Clinical review of the pediatric evaluation of disability Inventory. Br J Occup Ther (2001) 63(1):29–32. 10.1177/030802260006300106

[35] Chen KL Tseng MH Hu FC Koh CL. Pediatric evaluation of disability inventory: a cross-cultural comparison of daily function between Taiwanese and American children. Res Dev Disabil (2010) 31(6):1590–600. 10.1016/j.ridd.2010.05.002 20542661

[36] Berg M Jahnsen R Froslie KF Hussain A. Reliability of the pediatric evaluation of disability inventory (PEDI). Phys Occup Ther Pediatr (2004) 24(3):61–77. 10.1300/J006v24n03_05 15257969

[37] Iyer LV Haley SM Watkins MP Dumas HM. Establishing minimal clinically important differences for scores on the pediatric evaluation of disability inventory for inpatient rehabilitation. Phys Ther (2003) 83(10):888–98. 14519060

[38] Yeh CH Chen ML Li W Chuang HL. The Chinese version of the parenting stress index: a psychometric study. Acta Paediatr (2001) 90(12):1470–7. 10.1111/j.1651-2227.2001.tb01615.x 11853348

[39] King GA McDougall J Palisano RJ Grtizen J Tucker M Goal attainment scaling: its use in evaluating pediatric therapy programs. Phys Occup Ther Pediatr (1999) 19:31–52. 10.1080/J006v19n02_03

[40] Palisano RJ. Validity of goal attainment scaling in infants with motor delays. Phys Ther (1993) 73(10):651–8; discussion 8–60. 10.1093/ptj/73.10.651 7690976

[41] Steenbeek D Ketelaar M Galama K Gorter JW. Goal attainment scaling in paediatric rehabilitation: a report on the clinical training of an interdisciplinary team. Child Care Health Dev (2008) 34(4):521–9. 10.1111/j.1365-2214.2008.00841.x 19154553

[42] Rosenthal R An application of the Kolmogorov-Smirnov test for normality with estimated mean and variance. Psychol Rep (1968) 22(2):570 10.2466/pr0.1968.22.2.570 5650254

[43] Logan SW Feldner HA Bogart KR Goodwin B Ross SM Catena MA Toy-based technologies for children with disabilities simultaneously supporting self-directed mobility, participation, and function: a technical report. Front Robot AI (2017) 4:1–10. 10.3389/frobt.2017.00007

[44] Jones MA McEwen IR Hansen L. Use of power mobility for a young child with spinal muscular atrophy. Phys Ther (2003) 83:253–62. 12620089

[45] Livingstone R Field D. The child and family experience of power mobility: a qualitative synthesis. Dev Med Child Neurol (2015) 57(4):317–27. 10.1111/dmcn.12633 25403793

[46] Casey J Paleg G Livingstone R Facilitating child participation through power mobility. Br J Occup Ther (2013) 76:157–9. 10.4276/030802213X13627524435306

[47] Case-Smith J. Systematic review of interventions to promote social-emotional development in young children with or at risk for disability. Am J Occup Ther (2013) 67(4):395–404. 10.5014/ajot.2013.004713 23791314

[48] Lavigne JV Lebailly SA Gouze KR Binns HJ Keller J Pate L. Predictors and correlates of completing behavioral parent training for the treatment of oppositional defiant disorder in pediatric primary care. Behav Ther (2010) 41(2):198–211. 10.1016/j.beth.2009.02.006 20412885 PMC3522081

[49] Plueck J Freund-Braier I Hautmann C Beckers G Wieczorrek E Doepfner M. Recruitment in an indicated prevention program for externalizing behavior – parental participation decisions. Child Adolesc Psychiatry Ment Health (2010) 4:15. 10.1186/1753-2000-4-15 20509920 PMC2897776

[50] Cason J Behl D Ringwalt S. Overview of states’ use of telehealth for the delivery of early intervention (IDEA part C) services. Int J Telerehabil (2012) 42:39–46. 10.5195/IJT.2012.6105 25945202 PMC4296829

[51] Kierfeld F Ise E Hanisch C Gortz-Dorten A Dopfner M. Effectiveness of telephone-assisted parent-administered behavioural family intervention for preschool children with externalizing problem behaviour: a randomized controlled trial. Eur Child Adolesc Psychiatry (2013) 22(9):553–65. 10.1007/s00787-013-0397-7 23463180

[52] Svedberg LE Englund E Malker H Stener-Victorin E. Comparison of impact on mood, health, and daily living experiences of primary caregivers of walking and non-walking children with cerebral palsy and provided community services support. Eur J Paediatr Neurol (2010) 14(3):239–46. 10.1016/j.ejpn.2009.07.001 19628416

[53] Tefft D Guerette P Furumasu J. The impact of early powered mobility on parental stress, negative emotions, and family social interactions. Phys Occup Ther Pediatr (2011) 31(1):4–15. 10.3109/01942638.2010.529005 21080784

[54] Logan SW Ross SM Schreiber MA Feldner HA Lobo MA Catena MA Why we move: social mobility behaviors of non-disabled and disabled children across childcare contexts. Front Public Health (2016) 4:204. 10.3389/fpubh.2016.00204 27709110 PMC5030269

